# Genome-Scale CRISPR Screens Reveal DNA Repair Dependencies That Sensitize Hepatocellular Carcinoma to Oxaliplatin

**DOI:** 10.3390/cancers18091360

**Published:** 2026-04-24

**Authors:** Hanyue Ouyang, Diyun Huang, Dongsheng Wen, Lichang Huang, Zichao Wu, Zhicheng Lai, Minke He, Wenchao Wu, Ming Shi

**Affiliations:** 1 Department of Hepatobiliary Oncology, Sun Yat-sen University Cancer Center, Guangzhou 510060, China; ouyhy@sysucc.org.cn (H.O.); huangdy1@sysucc.org.cn (D.H.); wends@sysucc.org.cn (D.W.); huanglc1@sysucc.org.cn (L.H.); wuzc1@sysucc.org.cn (Z.W.); laizc@sysucc.org.cn (Z.L.); hemk@sysucc.org.cn (M.H.); 2 State Key Laboratory of Oncology in South China, Guangdong Provincial Clinical Research Center for Cancer, Sun Yat-sen University Cancer Center, Guangzhou 510060, China

**Keywords:** hepatocellular carcinoma, oxaliplatin, CRISPR-Cas9 screen, DNA repair

## Abstract

**Background**: Most patients with hepatocellular carcinoma (HCC) present with advanced disease and have limited systemic treatment options. Oxaliplatin shows clinical activity in HCC but its effectiveness is frequently curtailed by intrinsic and acquired resistance. We sought to systematically identify genetic vulnerabilities that increase oxaliplatin sensitivity in HCC. **Methods**: Genome-scale negative-selection CRISPR–Cas9 screens were conducted in two genetically distinct HCC cell lines (Hep3B and MHCC-97H) under low-dose oxaliplatin to discover conserved determinants of sensitivity. Selected DNA damage response (DDR) hits were validated. An oxaliplatin-resistant MHCC-97H subline was generated for transcriptomic profiling to characterize resistance-associated programs. Screen results were integrated with TCGA-LIHC expression and survival data to evaluate clinical relevance. Additionally, we analyzed bulk RNA-seq data from biopsy specimens collected from 36 HCC patients prior to initiation of hepatic arterial infusion chemotherapy (HAIC), comparing expression levels of the DDR genes between patients with objective response and non-responders. **Results**: Screens in both cell lines converged on DDR pathways, particularly nucleotide excision repair (NER) and the Fanconi anemia/interstrand crosslink repair network; shared sensitizers included *ERCC4* (*XPF*), *FANCE* and *SLX4*. Validation experiments showed that disruption of representative DDR factors (*POLH* and *XPA*) synergistically increased oxaliplatin efficacy at concentrations as low as 0.5 μM. Transcriptomic analysis of the resistant MHCC-97H subline revealed coordinated upregulation of DNA repair programs, G2/M checkpoint and E2F target signatures, and epithelial–mesenchymal transition features. Integration with TCGA-LIHC data demonstrated frequent overexpression of many screen-identified DDR genes in primary HCC and an association between higher expression of selected factors and poorer patient survival. In the HAIC cohort, several DDR genes, including *ATR*, *BRCA2*, *CDK7*, *MUS81*, *MUTYH*, *PARG*, *POLH*, *POLK* and *XPA*, were significantly lower in the objective response group. **Conclusions**: DDR components represent candidate biomarkers and therapeutic targets whose inhibition may enhance oxaliplatin efficacy in HCC.

## Simple Summary

Hepatocellular carcinoma is the sixth-most common cancer and the third-leading cause of cancer-related death. Hepatic arterial infusion chemotherapy with a FOLFOX regimen which contains oxaliplatin has been demonstrated to provide survival benefit, but chemoresistance substantially reduces response rates and clinical efficacy. To systematically identify strategies that increase oxaliplatin sensitivity, we performed genome-scale negative-selection CRISPR-Cas9 screens in two hepatocellular carcinoma cell lines. DNA damage response pathways emerged as conserved determinants of sensitivity to oxaliplatin, and most screen-identified DNA repair genes are frequently overexpressed in primary hepatocellular carcinoma, and higher expression of selected genes is associated with poorer patient survival. DNA repair components may act as candidate biomarkers and therapeutic targets to improve oxaliplatin outcomes in liver cancer.

## 1. Introduction

Hepatocellular carcinoma (HCC) remains one of the leading causes of cancer mortality worldwide, and effective systemic therapies for advanced disease are limited [1,2,3]. Oxaliplatin, a third-generation platinum compound used in regimens such as FOLFOX, has demonstrated clinical activity and improved survival in HCC [4,5]. Moreover, oxaliplatin-based hepatic artery infusion chemotherapy (HAIC), alone or in combination, has shown benefit in selected patients, supporting a potential role for oxaliplatin in HCC treatment [6,7,8]. Nevertheless, response rates and durability of benefit remain suboptimal, because therapeutic efficacy is frequently constrained by intrinsic and acquired resistance to platinum agents [9,10,11,12,13,14]. Identifying strategies that increase oxaliplatin sensitivity is therefore essential for improving patient outcomes [15].

Platinum drugs exert cytotoxicity primarily by forming platinum–DNA adducts, most commonly intrastrand crosslinks between adjacent purines and, less frequently but often more deleteriously, interstrand crosslinks (ICLs) that covalently join opposite DNA strands [16,17,18]. These lesions impede transcription and replication, provoke replication-fork stalling and double-strand breaks, and activate a networked DNA damage response (DDR) that dictates cell-cycle arrest, repair or cell death. Multiple mechanisms of resistance have been described, including altered drug accumulation, enhanced drug efflux, factors that prevent drug–target interaction, and pathways that remove DNA damage [17,18,19,20,21]. Although individual factors have been implicated in platinum resistance in different cancers, a systematic, unbiased investigation of sensitizers that enhance the efficacy of oxaliplatin in HCC remains lacking.

We aimed to systematically identify candidates that act synergistically with low-dose oxaliplatin in HCC. To this end, we performed genome-scale negative-selection CRISPR screens in two genetically distinct HCC models (Hep3B and MHCC-97H) under low-dose oxaliplatin. Integrative analysis between two cell lines revealed a conserved reliance on DDR pathways—notably, nucleotide excision repair (NER) and the Fanconi anemia/ICL repair network. Representative DDR hits (the translesion synthesis polymerase *POLH* and the nucleotide excision repair factor *XPA*) were validated by competitive growth and colony formation assays in Hep3B cells and were confirmed to enhance oxaliplatin efficacy synergistically at oxaliplatin concentrations as low as 0.5 μM.

To model acquired resistance, we derived an oxaliplatin-resistant MHCC-97H subline and performed transcriptomic profiling. Resistant cells showed coordinated upregulation of DNA repair programs, G2/M checkpoint and E2F target signatures, and epithelial–mesenchymal transition (EMT) features, consistent with a multifaceted resistance phenotype. Integration with TCGA-LIHC expression data demonstrated that most screen-identified DDR genes are overexpressed in primary HCC and that high expression of selected factors correlates with poorer patient survival. In the HAIC cohort, several DDR genes were significantly lower in the objective response group, including *ATR*, *BRCA2*, *CDK7*, *MUS81*, *MUTYH*, *PARG*, *POLH*, *POLK* and *XPA*. In summary, these results identify DDR components as conserved determinants of sensitivity that cooperate with oxaliplatin, suggesting that their inhibition could enhance oxaliplatin sensitivity in HCC.

## 2. Materials and Methods

### 2.1. Cell Lines and Culture

Hep3B and MHCC-97H hepatocellular carcinoma cell lines (GuangZhou Jennio Biotech Co., Ltd., Guangzhou, China) were cultured in DMEM (Gibco, Thermo Fisher Scientific, Waltham, MA, USA) supplemented with 10% fetal bovine serum (FBS; Sigma-Aldrich, St. Louis, MO, USA; cat. no. F8318) and 1% penicillin/streptomycin (Pen/Strep; Thermo Fisher Scientific, Waltham, MA, USA) at 37 °C in 5% CO_2_. Cell lines were authenticated by short tandem repeat (STR) profiling. The 293T packaging cell line (ATCC CRL-3216; ATCC, Manassas, VA, USA) was used for lentiviral production.

### 2.2. Lentivirus Production of Human CRISPR KO GeCKO v2.0 Library A + B

Lentiviral particles were produced in 293T cells grown in 180 mm dishes to ~70% confluence. Transfections were performed using 54 µL of FugeneHD (Promega, Madison, WI, USA; catalog no. E2311) with a plasmid mixture consisting of 6 µg of VSV-G, 6 µg of PAX2, 3 µg of CRISPR KO Library A, and 3 µg of CRISPR KO Library B (Addgene, Watertown, MA, USA; Addgene plasmid #1000000049). Culture medium was refreshed 24 h post-transfection and viral supernatant collected 48 h later, filtered through a 0.45 µm filter, aliquoted and stored at −80 °C.

### 2.3. Genome-Wide CRISPR Knockout Screening in Hep3B and MHCC-97H Cell Lines

Hep3B and MHCC-97H cells stably expressing Streptococcus pyogenes Cas9 (Hep3B + Cas9 and MHCC-97H + Cas9) were generated prior to screening. For each screen, cells were transduced with the GeCKO v2 pooled sgRNA library (A + B) at a multiplicity of infection (MOI) of 0.3 to favor single-integrant events. Forty-eight hours after transduction, cells were selected with puromycin (2 µg/mL; Sigma-Aldrich, St. Louis, MO, USA) for 3 days. After selection, an aliquot of cells was harvested as the baseline sample (Day 0). Cells were then split and cultured for 8 days in either 1 µM oxaliplatin (Jiangsu Hengrui Pharmaceuticals Co., Ltd., Lianyungang, China) or vehicle control (DMSO). Media and drug were refreshed every 48 h. At the end of the treatment period (Day 8), genomic DNA was harvested for sgRNA abundance quantification.

Genomic DNA was extracted using a Zymo Research Quick-gDNA MidiPrep kit (Zymo Research, Irvine, CA, USA) according to the manufacturer’s protocol from cells to maintain >500× library coverage (minimum 6 × 10^7^ cells per sample). sgRNA cassettes were PCR-amplified using NEBNext High-Fidelity PCR Master Mix (New England Biolabs, Ipswich, MA, USA); multiple PCR reactions per sample were pooled, gel-purified and combined to generate sequencing libraries. Libraries were sequenced on an Illumina platform (Illumina, San Diego, CA, USA), targeting ~60 million reads per sample to ensure deep coverage of the pooled library.

Demultiplexing and mapping of reads to the reference sgRNA library, and normalization and quality control, were performed with MAGeCK (v0.5.9). Read counts were normalized across samples; QC metrics included the Gini index (threshold < 0.2), the fraction of zero-count sgRNAs and overall library coverage (>99%), as reported. Gene-level negative-selection rankings were generated using the MAGeCK RRA algorithm (MAGeCK v0.5.9). To identify candidate oxaliplatin sensitizers, comparisons were performed between oxaliplatin-treated and DMSO control samples.

### 2.4. Plasmid Constructs and Generation of Knockout and Gene-Activation Cell Lines

Hep3B and MHCC-97H cells were transduced with pLv5-Cas9-Neo to establish stable Cas9-expressing lines (Hep3B + Cas9 and MHCC-97H + Cas9) and were selected with G418 for 10 days. To generate *POLH* and *XPA* knockouts, target-specific single-guide RNAs (sgRNAs) were cloned into the doxycycline-inducible FgH1tUTG vector (Addgene #70183), which co-expresses EGFP for selection. Hep3B + Cas9 and MHCC-97H + Cas9 cells were transduced with the FgH1tUTG-sgRNA constructs; sgRNA expression was induced by doxycycline (2 µg/mL; Sigma-Aldrich, St. Louis, MO, USA) for 3 days, and EGFP-positive cells were enriched by fluorescence-activated cell sorting (FACS, BD FACSAria II, BD Biosciences, San Jose, CA, USA) to obtain pooled, sgRNA-expressing populations.

For *TP73* activation, Hep3B and MHCC-97H cells were transduced with a lentiviral vector encoding the dCas9-VP64 fusion protein to establish stable dCas9-VP64-expressing lines. Stable integrants were obtained by antibiotic selection. To activate *TP73*, promoter-targeting sgRNAs were cloned into the pXPR_502-sgRNA plasmid (Addgene, Watertown, MA, USA). To enable convenient enrichment of sgRNA-expressing cells, pZIP-SFFV-GFP lentivirus was co-packaged and co-transduced with pXPR_502-sgRNA virus. Seventy-two hours after infection, GFP-positive cells were isolated by fluorescence-activated cell sorting (FACS) to obtain pooled, sgRNA-expressing populations with activated *TP73*.

The sgRNA sequences used were:

*XPA*, 5′-GTTGGGCTTGTTTAGTCCAC-3′;

*POLH*, 5′-TATGTAATATGCGAAGAATG-3′;

*ERCC4*, 5′-gCCATAACCCATCGCTTGAAG-3′;

*FANCE*, 5′-GAGACCCGAACATAAGTCAC-3′;

*SLX4*, 5′-gTTCAGAAGAGTGCTCCCTCG-3′;

Non-target Control, 5′-GTATTACTGATATTGGTGGG-3′;

*TP73*, 5′-gCGGGCCACCGAGTCGCGGCG-3′.

### 2.5. Competitive (Mixing) Assay by Flow Cytometry

GFP-positive, sgRNA-transduced cells were mixed 1:1 with GFP-negative parental Hep3B and MHCC-97H cells, cultured with DMSO or oxaliplatin. After days of treatment, cells were harvested, passed through a 40 µm strainer (Falcon; Corning, Corning, NY, USA) to obtain a single-cell suspension and analyzed on a flow cytometer (BD LSRFortessa; BD Biosciences, San Jose, CA, USA). Live singlets were gated using FSC/SSC, and GFP thresholds were set using GFP-negative controls. The fraction of GFP+ cells was recorded for each condition.

### 2.6. Colony Formation Assay

For colony assays, control and knockout cells were seeded in 6-well plates (seeding densities: *POLH*, 5000 cells/well; *XPA*, 2000 cells/well), treated with DMSO or oxaliplatin (0.5 µM, 1 µM) and incubated for 10 days with medium changes every 3–4 days. Colonies were fixed with 4% paraformaldehyde (Beyotime Biotechnology, Shanghai, China), stained with 0.5% crystal violet (Beyotime Biotechnology, Shanghai, China), washed, air-dried and quantified. Colonies > 50 cells were counted manually or by image analysis; values were normalized to the corresponding DMSO control.

### 2.7. Generation of Oxaliplatin-Resistant MHCC-97H Subline

An oxaliplatin-resistant MHCC-97H derivative was generated by continuous culture under gradually increasing, sublethal concentrations of oxaliplatin. Cells were initially exposed to a low dose and, upon stabilization of growth, the drug concentration was increased stepwise over weeks to months until a resistant population was obtained. Resistance was confirmed by increased IC50 relative to parental MHCC-97H. Cell line identity was verified by short tandem repeat (STR) profiling analysis using the AmpFLSTR Identifiler kit (Thermo Fisher Scientific, Waltham, MA, USA) according to the manufacturer’s instructions. Resulting STR profiles for the oxaliplatin-resistant subline were compared with the parental MHCC-97H profile and public reference databases; the resistant subline matched the parental MHCC-97H profile. We acknowledge that, beyond STR verification of cell line identity, we did not perform comprehensive genomic or epigenetic profiling to assess the stability of the transcriptional changes observed.

### 2.8. Cell Viability and IC50 Determination

For dose–response studies and IC50 determination, cells were seeded in 96-well plates (triplicate wells per condition). Eight hours after seeding, serial dilutions of oxaliplatin were added. Cell viability was measured 48 h or 72 h after treatment using the CCK-8 assay (CCK-8; Dojindo Molecular Technologies, Kumamoto, Japan) according to the manufacturer’s instructions; absorbance was read on a microplate reader. IC50 values were calculated from nonlinear regression fits of dose–response curves.

### 2.9. Immunofluorescence (IF) and Confocal Microscopy

Cells cultured on a coverslip were washed once with PBS and fixed in 3.7% formalin containing 1% methanol for 10 min at room temperature. After washing three times with PBS, samples were incubated 30 min in PBS containing 10% normal goat serum (Jackson ImmunoResearch, West Grove, PA, USA), 0.2% fish skin gelatin (Sigma-Aldrich, St. Louis, MO, USA) and 0.1% Triton X-100 (PBS-NGS). Next, samples were incubated with primary antibodies diluted in PBS-NGS for 1 h, rinsed extensively with PBS containing 0.1% Triton X-100 for 30 min, and then incubated with Alexa Fluor 555-conjugated secondary antibodies (Thermo Fisher Scientific, Waltham, MA, USA) diluted in PBS-NGS for 40 min at room temperature. Following additional extensive rinsing for 30 min, samples were stained for 15 min DAPI (Beyotime Biotechnology, Shanghai, China) diluted 1:500 in PBS to detect nuclei. After final rinsing in PBS, samples were mounted with FluorSave (Merck Millipore, Burlington, MA, USA).

### 2.10. Host Cell Reactivation Assay

PLVX-EF1α-mCherry plasmid DNA (TaKaRa Bio Inc., Kusatsu, Shiga, Japan) was was incubated with 20 µM oxaliplatin in PBS at 37 °C overnight to introduce platinum–DNA adducts, and plasmid DNA incubated in PBS as an undamaged control. After incubation, plasmids were purified to remove unbound oxaliplatin by ethanol precipitation, quantified and normalized prior to transfection. Hep3B parental line, *POLH* and *XPA* knockout derivatives were seeded to reach ~60–80% confluence and transfected with equal amounts of either oxaliplatin-treated or untreated PLVX-EF1α-mCherry plasmid; undamaged PLVX-EF1α-mCherry plasmid was transfected in parallel as a reference using the same cell number and transfection conditions. Seventy-two hours after transfection, cells were harvested, gated for single, live cells, and analyzed by flow cytometry (BD LSRFortessa; BD Biosciences, San Jose, CA, USA) to determine the percentage of mCherry-positive cells. Repair efficiency was calculated as (% mCherry+ from oxaliplatin-treated plasmid/% mCherry+ from untreated plasmid) × 100. Experiments were performed in biological triplicate (*n* ≥ 3) and data are reported as mean ± SD; statistical comparisons used two-tailed *t*-tests, with *p* < 0.05 considered significant.

### 2.11. HAIC Patient Specimen Collection

Thirty-six patients with hepatocellular carcinoma who received hepatic arterial infusion chemotherapy (HAIC) at our institution between January 2016 and December 2023 were enrolled. The study protocol was reviewed and approved by the institutional review board and was conducted in accordance with the Declaration of Helsinki and applicable regulations. Written informed consent was obtained from all participants prior to biopsy for collection and research use of tumor tissue, including genomic and transcriptomic analyses; where use of archived specimens without written consent was necessary, this was permitted by the approving ethics committee. Percutaneous tumor biopsies were obtained under ultrasound guidance before initiation of HAIC to ensure accurate sampling and to minimize patient discomfort and risk. Collected biopsy specimens were immediately placed into RNase-free tubes; surface blood and gross contaminants were removed, and tissues were submerged in RNAlater stabilization solution (Ambion Inc., Austin, TX, USA) to preserve RNA integrity. Samples were held at 4 °C overnight to allow RNAlater penetration and then transferred to long-term storage at −80 °C. Total RNA was extracted from all specimens and subjected to rigorous quality control, and samples that met predefined quality thresholds were submitted for bulk RNA sequencing (RNA-seq).

### 2.12. RNA Extraction, Library Construction and RNA-Seq Analysis

Total RNA was extracted using TRIzol reagent (Ambion Inc., Austin, TX, USA) and quality-checked by spectrophotometry (NanoDrop 2000, Thermo Fisher Scientific, Waltham, MA, USA) and electrophoresis. Poly(A)-selected libraries were prepared from high-quality RNA and sequenced on an Illumina platform (Illumina, San Diego, CA, USA) to generate paired-end reads. Reads were quality controlled, aligned to the human reference genome and quantified to obtain gene-level counts. Differential expression analysis was performed with DESeq2 (DESeq2 v1.34.0). Pathway analyses included GSVA (hallmark gene sets, GSVA v1.44.0, Bioconductor) and GSEA (GSEA v4.2.3, Broad Institute, Cambridge, MA, USA) to identify enriched biological programs in resistant versus parental cells. Principal component analysis (PCA) was used to assess sample clustering.

### 2.13. Functional Enrichment and Network Analysis

Gene ontology (GO), KEGG and pathway enrichment analyses were performed on screen hits and differentially expressed gene sets. Protein–protein interaction networks and functional enrichment for union and cell line-specific hit sets were analyzed using STRING (STRING Consortium; EMBL-EBI, Hinxton, UK).

### 2.14. TCGA Data Analysis and Survival Analysis

TCGA-LIHC gene expression and clinical data were obtained via the Xena Browser (UCSC Xena, University of California Santa Cruz, Santa Cruz, CA, USA). Expression comparisons between tumor and normal liver samples were performed using appropriate statistical tests (*t*-test for cohort comparisons; paired t-test for matched tumor-adjacent normal pairs). For survival analyses of each DDR gene, patients were dichotomized into high- and low-expression groups by two approaches: (1) an optimal cutpoint selected by the minimum log-rank *p*-value method, and (2) a predefined median split. Kaplan–Meier OS curves were generated and group differences assessed by the log-rank test. Univariate Cox proportional hazards models were fitted for each gene; genes with nominal *p* < 0.05 in univariate analysis were further evaluated in multivariate Cox models that included available clinical covariates (age, sex and tumor stage).

### 2.15. Statistical Analysis

Statistical analyses were performed in R (R v4.1.2). MAGeCK (MAGeCK v0.5.9) was used for CRISPR screen normalization and RRA ranking. Differential expression of RNA-seq data was performed with DESeq2 (DESeq2 v1.34.0). GSVA v1.44.0 and GSEA (GSEA v4.2.3, Broad Institute, Cambridge, MA, USA) were used for pathway analyses. For survival analyses, the survminer v0.4.9 and survival packages v3.8-6 were used to compute optimal cutpoints and Kaplan–Meier estimates. For comparison tests, *t*-tests were applied unless stated otherwise. Significance thresholds are specified in the figure legends and Section 2; exact *p*-values are provided in the Figures.

## 3. Results

### 3.1. Genome-Scale Negative-Selection CRISPR-Cas9 Screen in Hep3B and Quality Control

**Figure 1:**
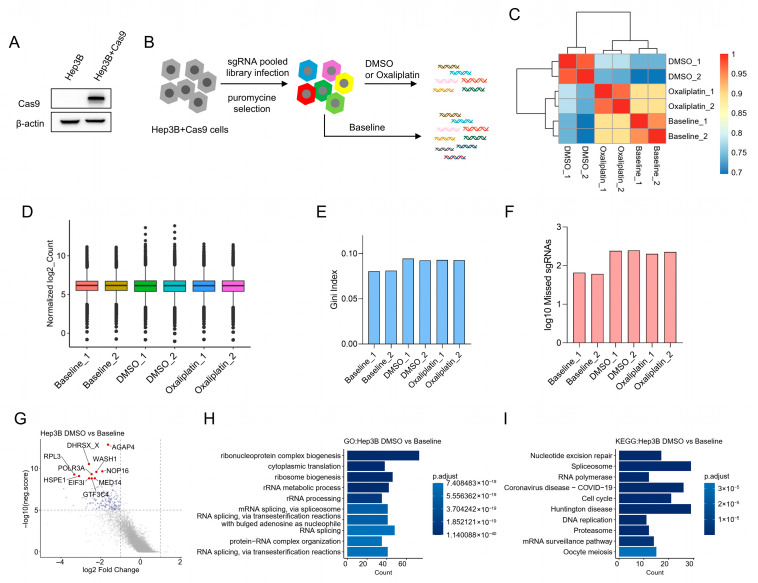
Genome-wide loss-of-function CRISPR-Cas9 screen in Hep3B cells. (**A**) Immunoblot confirming Cas9 expression in parental Hep3B (wild type) and Hep3B cells stably expressing Cas9 (Hep3B + Cas9). β-actin is shown as a loading control. (**B**) Experimental workflow: Hep3B + Cas9 cells were transduced at MOI = 0.3 with the GeCKO v2 pooled sgRNA library (A + B; Addgene #1000000049). After 72 h puromycin selection, baseline samples were collected (Day 0). Cells were then cultured for 8 days in either 1 μM oxaliplatin or DMSO (vehicle control), and samples were harvested on Day 8. Media and drug were refreshed every 48 h. Two independent biological replicates were performed per condition. (**C**) Pairwise Pearson correlation heatmap illustrating high concordance between biological replicates. (**D**) Distribution of normalized sgRNA read counts in each sample (normalization performed using MAGeCK). (**E**) Gini index for sgRNA read count distributions in each sample, indicating even representation across the library (values < 0.2). (**F**) Number of missing (zero-read) sgRNAs in each sample; overall library coverage exceeded 99% across samples. (**G**) MAGeCK gene-level summary comparing DMSO (Day 8) versus Baseline (Day 0): average sgRNA log2 fold-change per gene is plotted against −log10(neg.score). Genes significantly depleted in the DMSO control (−log10(neg.score) > 5 and log2 fold-change < −1) are highlighted in blue; the top 10 most depleted genes are indicated in red. (**H**,**I**) Gene ontology (**H**) and KEGG pathway (**I**) enrichment analyses performed on the gene set identified in (**G**). The original Western blot figures can be found in Supplementary Figures S6–S8.

To identify genes that increase oxaliplatin sensitivity in HCC, we performed an unbiased, genome-scale negative-selection (drop-out) CRISPR-Cas9 screen in the Hep3B hepatocellular carcinoma cell line. Isogenic Hep3B cells stably expressing Cas9 (Hep3B + Cas9) were generated (Figure 1A) and transduced with the GeCKO v2.0 library. After puromycin selection, genomic DNA was collected at baseline (day 0) and after 8 days of treatment with low-dose oxaliplatin (1 μM) or DMSO (vehicle control), and sgRNA abundance was quantified by next-generation sequencing (Figure 1B).

To define screening conditions, we first performed dose–response viability assays in Hep3B and MHCC-97H cell lines; representative curves are shown in Supplementary Figure S1. Screening concentrations were chosen to balance two competing requirements of negative-selection screens: (i) a concentration sufficient to impose selective pressure so that loss of sensitizing genes leads to guide dropout, and (ii) a sublethal exposure that preserves overall population size and library representation to avoid bottlenecks. The dose–response analysis indicated that 1.0 μM oxaliplatin corresponded approximately to the IC10-IC20 range in the tested lines; therefore, 1.0 μM was selected because it produced a reproducible, modest reduction in viability over the assay interval while allowing maintenance of sufficient cell numbers and doublings to preserve library complexity.

Two biological replicates were obtained per condition, and replicate concordance was high (Figure 1C). We applied several quality-control metrics to assess screen performance. Read counts were normalized across samples using MAGeCK (Figure 1D). Gini indices across samples were below 0.2, indicating an even sgRNA count distribution appropriate for negative selection analysis (Figure 1E). The fraction of sgRNAs with zero counts was very low and overall library coverage exceeded 99% (Figure 1F). At the gene level, DMSO control screens showed strong depletion of sgRNAs targeting core essential genes; the most depleted genes were significantly enriched for essential processes such as translation, ribosome biogenesis, RNA splicing, and cell cycle regulation (Figure 1G–I).

### 3.2. CRISPR Screen Identifies DNA Repair Genes That Sensitize Hep3B Cells to Oxaliplatin

**Figure 2:**
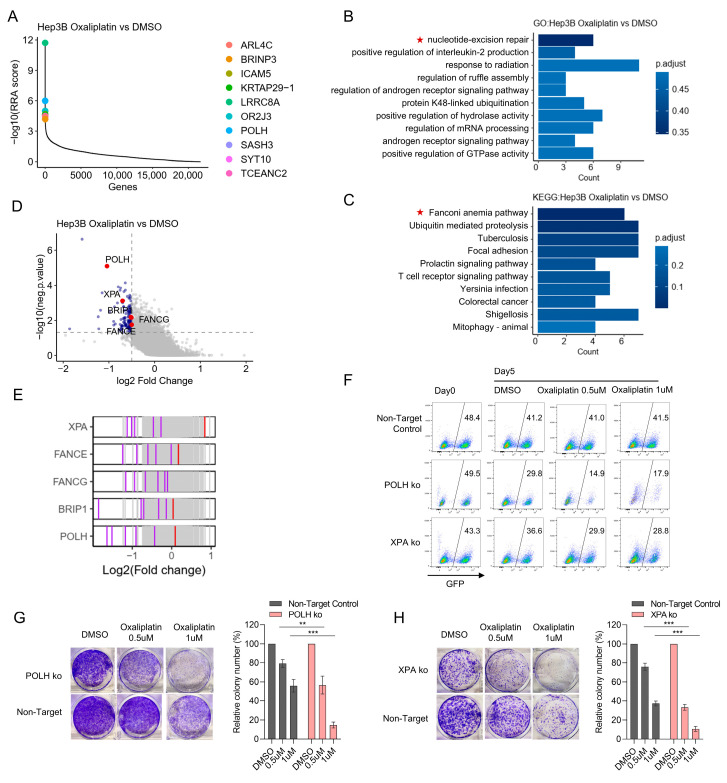
Validation of CRISPR-identified DNA repair genes whose loss increases Hep3B cell sensitivity to oxaliplatin. (**A**) MAGeCK RRA gene-rank plot comparing oxaliplatin (Day 8) versus DMSO (Day 8); the top 10 significantly negatively selected genes (lowest RRA scores) are listed. (**B**,**C**) Enrichment analyses for genes significantly depleted in oxaliplatin-treated samples: Gene ontology (biological process (**B**)) and KEGG pathways (**C**). Thresholds for inclusion were *p* value < 0.05 and log2 fold-change < −0.5. Red stars indicates signaling pathways related to DDR pathways. (**D**) Volcano plot indicating genes depleted upon oxaliplatin treatment; selected DNA-repair genes are highlighted in red. (**E**) sgRNA-level effects for representative DNA-repair genes identified in the screen. Each vertical tick represents an individual sgRNA plotted at its log2 fold-change; consistent depletion across independent sgRNAs supports on-target activity. (**F**) Competitive growth (mixing) assay validating *POLH* and *XPA* hits. GFP tagged sgRNA-transduced Hep3B + Cas9 cells (non-targeting control, *POLH*, *XPA*) were mixed 1:1 with GFP- parental Hep3B (wt) cells, treated with DMSO or oxaliplatin (0.5 μM, 1 μM), and analyzed by flow cytometry after 5 days. Plotted values show the fraction of GFP+ cells in each condition. (**G**,**H**) Colony-formation assays validating *POLH* (**G**) and *XPA* (**H**). Non-targeting control and knockout cells were seeded (5000 cells/well for *POLH*; 2000 cells/well for *XPA*) in 6-well plates, treated for 10 days with DMSO or oxaliplatin (0.5 μM, 1 μM), stained with crystal violet, and quantified. Representative wells are shown at left; relative colony counts (normalized to respective DMSO controls) are plotted at right. Statistical data are presented as mean ± SD. Statistical testing was performed using an unpaired two-tailed Student’s *t*-test; *p*-values are shown. Significance is indicated as: ** *p* < 0.01; *** *p* < 0.001.

To identify genes whose loss selectively sensitizes Hep3B cells to oxaliplatin, we compared oxaliplatin-treated samples to DMSO controls. Genes were ranked by negative selection using the MAGeCK RRA algorithm; the top ten depleted genes are shown in Figure 2A. Pathway enrichment analysis of genes significantly depleted in oxaliplatin-treated samples revealed strong enrichment for nucleotide excision repair (NER) and the Fanconi anemia (FA) pathway (Figure 2B,C). Notably, multiple DNA damage response genes were reproducibly depleted, with at least two independent sgRNAs showing consistent depletion per gene; representative hits include the translesion synthesis polymerase *POLH*, the NER factor *XPA*, and FA pathway components *FANCA*, *FANCG* and *BRIP1* (Figure 2D,E).

To validate the screen, we targeted *POLH* and *XPA* using sgRNAs derived from the GeCKO v2.0 library, with a non-targeting sgRNA as a negative control. GFP-labeled, sgRNA-transduced Hep3B cells were mixed 1:1 with wild-type cells and cultured in DMSO or low-dose oxaliplatin (0.5 µM and 1 µM). After 5 days, the GFP+ fraction harboring *POLH*- or *XPA*-targeting sgRNAs was selectively depleted under oxaliplatin treatment compared with DMSO, whereas the non-targeting sgRNA control did not show this decline, indicating reduced survival of *POLH* and *XPA* knockout cells in the presence of drug (Figure 2F). Colony formation assays further confirmed that loss of *POLH* or *XPA* sensitizes Hep3B cells to oxaliplatin (Figure 2G,H).

### 3.3. POLH/XPA Deficiency Increases γH2AX and Impairs Repair of Oxaliplatin-Induced DNA Damage

**Figure 3:**
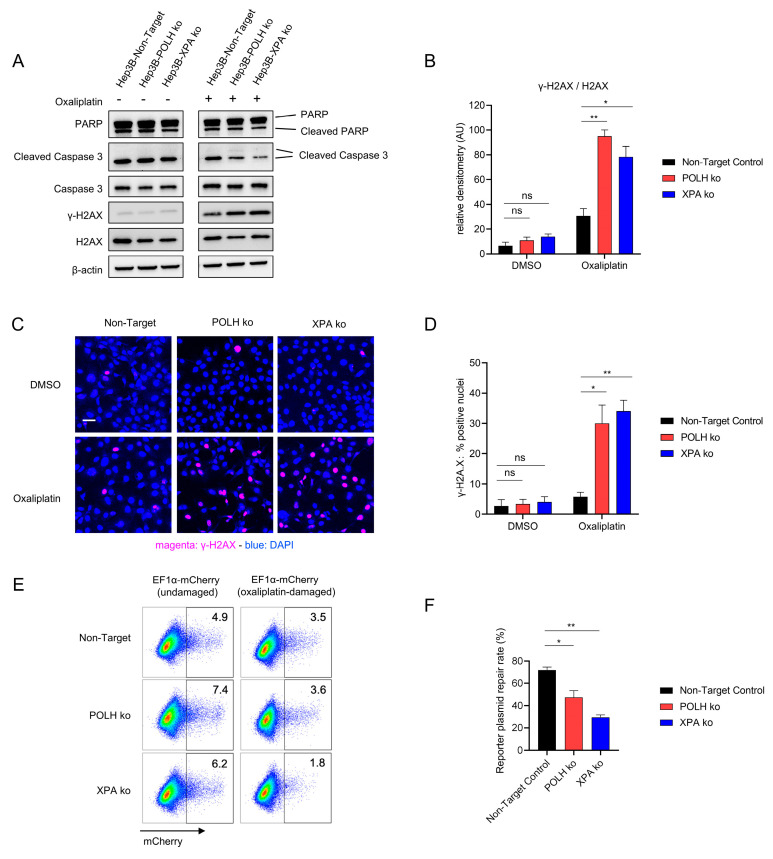
Loss of *POLH* or *XPA* increases oxaliplatin-induced DNA damage and reduces repair of oxaliplatin-damaged DNA. (**A**) Representative Western blots for γH2AX, cleaved caspase-3, total caspase-3, PARP and β-actin (loading control) in non-target control and *POLH*- or *XPA*-knockout Hep3B cells treated with DMSO or 20 μM oxaliplatin for 24 h. (**B**) Quantification of γH2AX signal normalized to total H2AX (mean ± SD, *n* = 3 independent experiments). Two-tailed Student’s t-tests were used for pairwise comparisons. * *p* < 0.05; ** *p* < 0.01; ns, not significant. (**C**) Immunofluorescence staining for γH2AX (red) and DAPI (blue) in control and *POLH*- or *XPA*-deficient cells treated with DMSO or 20 μM oxaliplatin for 24 h. Representative images are shown (scale bar = 10 μm). (**D**) Quantification of the percentage of γH2AX-positive nuclei (mean ± SD, *n* = 3 fields per condition from three independent experiments). Statistical comparisons were performed using two-tailed Student’s t-tests; * *p* < 0.05; ** *p* < 0.01; ns, not significant. (**E**) Representative flow cytometry dot plots showing PLVX-EF1α-mCherry reporter expression in cells transfected with either undamaged or oxaliplatin-treated reporter plasmid. Plasmid DNA was incubated with 20 μM oxaliplatin in PBS at 37 °C overnight to generate platinum–DNA adducts, while control plasmid was incubated in PBS without drug. Plasmids were purified by ethanol precipitation to remove unbound oxaliplatin, then quantified, normalized, and transfected into cells. Seventy-two hours after transfection, single, live cells were gated and analyzed by flow cytometry to determine the percentage of mCherry-positive cells; numbers in the plots indicate the % mCherry+ cells. (**F**) Quantification of reporter plasmid recovery, calculated as (% mCherry+ from oxaliplatin-treated plasmid/% mCherry+ from untreated plasmid) × 100 (mean ± SD, *n* = 3 independent transfections). Two-tailed Student’s *t*-tests were used for statistical comparisons; * *p* < 0.05; ** *p* < 0.01. The original Western blot figures can be found in Supplementary Figure S9.

To investigate how loss of *POLH* or *XPA* enhances oxaliplatin sensitivity, we measured markers of DNA damage and apoptosis. After oxaliplatin treatment, *POLH*- and *XPA*-deficient Hep3B cells exhibited markedly higher γH2AX levels by Western blot when normalized to total H2AX, whereas increases in cleaved caspase-3 and cleaved PARP were minimal and not significant at the tested time point (Figure 3A,B). Immunofluorescence confirmed a greater proportion of γH2AX-positive nuclei in *POLH-* and *XPA*-deficient cells following oxaliplatin exposure (Figure 3C,D).

Direct assessment of repair using an oxaliplatin-treated PLVX-EF1A-mCherry reporter plasmid showed reduced recovery of reporter expression in *POLH*- and *XPA*-deficient cells compared with non-target controls, consistent with impaired removal of platinum-induced DNA lesions (Figure 3E,F). Together, these results indicate that loss of *POLH* or *XPA* increases oxaliplatin sensitivity primarily by compromising the cellular ability to resolve platinum-induced DNA damage rather than by substantially increasing apoptosis under the sublethal conditions used in the screen.

### 3.4. Integrated CRISPR Screens Identify Conserved DNA Repair Determinants of Oxaliplatin Sensitivity in Hep3B and MHCC-97H Cells

We next asked which oxaliplatin sensitizers are conserved across hepatocellular carcinoma models, since CRISPR screen results can be cell-line dependent due to differences in genetic background, DDR capacity, cell-cycle distribution, and drug handling. To address this, we performed another independent CRISPR screen in a second hepatocellular carcinoma line, MHCC-97H.

**Figure 4:**
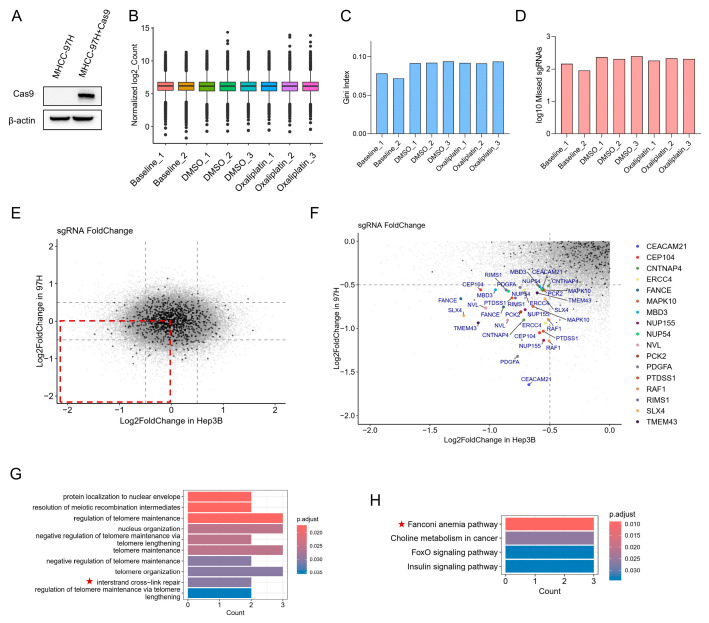
Integrated CRISPR screens identify conserved DNA-repair determinants of oxaliplatin sensitivity in Hep3B and MHCC-97H cells. (**A**) Immunoblot confirming Cas9 expression in parental MHCC-97H (WT) and MHCC-97H cells stably expressing Cas9 (MHCC-97H + Cas9). β-actin is shown as a loading control. (**B**) Distribution of normalized sgRNA read counts for each sample (normalization performed with MAGeCK). Two replicates were obtained for the baseline sample, and three biological replicates were obtained for each of the DMSO and oxaliplatin conditions. (**C**) Gini index of the sgRNA read count distributions for each sample. (**D**) Number of missing (zero-read) sgRNAs in each sample. (**E**) Scatterplot of sgRNA log2 fold-changes (oxaliplatin vs. DMSO) in Hep3B (x axis) versus MHCC-97H (y axis, MHCC-97H is abbreviated as “97H” in the figure). The red box indicates the region of concordant depletion in both cell lines. (**F**) Magnified view of the boxed region in (**E**). Genes represented by at least two independent sgRNAs depleted in both screens (log2 fold-change < −0.5 in both cell lines) are highlighted; using this criterion we identified 17 overlapping hits, including *ERCC4 (XPF)*, *FANCE* and *SLX4.* (**G**,**H**) Gene ontology (**G**) and KEGG pathway (**H**) enrichment analyses performed on the shared gene set from (**F**), showing significant enrichment for interstrand cross-link repair and the Fanconi anemia pathway. Red stars indicates signaling pathways related to DDR pathways. The original Western blot figures can be found in Supplementary Figures S6–S8.

We first generated a stable MHCC-97H cell line expressing Cas9 (MHCC-97H + Cas9) (Figure 4A) and carried out the screen using the same experimental pipeline as for Hep3B. Library quality and screen performance were verified by standard metrics: total read counts, Gini index, and the fraction of sgRNAs with zero counts, all of which indicated satisfactory representation and coverage (Figure 4B–D).

We then computed fold-changes for all sgRNAs under oxaliplatin treatment and examined the overlap between Hep3B and MHCC-97H (red boxed region in Figure 4E; MHCC-97H is abbreviated as “97H” in the figure). To define gene-level hits, we required ≥2 independent sgRNAs per gene to be depleted in both screens in oxaliplatin-treated samples (log2 fold-change < −0.5 and *p*-value < 0.05 in both cell lines). Using this threshold, we identified 17 overlapping hits, including the NER endonuclease *ERCC4* (*XPF*) and Fanconi anemia pathway components *FANCE* and *SLX4* (Figure 4F). Pathway enrichment analysis using GO and KEGG annotations on the shared depleted genes revealed a strong overrepresentation of interstrand cross-link repair (ICL) and the Fanconi anemia pathway (Figure 4G,H), indicating that these pathways are conserved determinants of oxaliplatin sensitivity across HCC cell lines. Telomere maintenance-related terms were also highlighted, consistent with the well-established roles of DDR proteins at telomeres: defects in these factors can lead to telomere shortening or dysfunction, chromosome fusions and consequent genomic instability [22,23].

### 3.5. Establishment and Transcriptomic Profiling of an Oxaliplatin-Resistant MHCC-97H Subline

**Figure 5:**
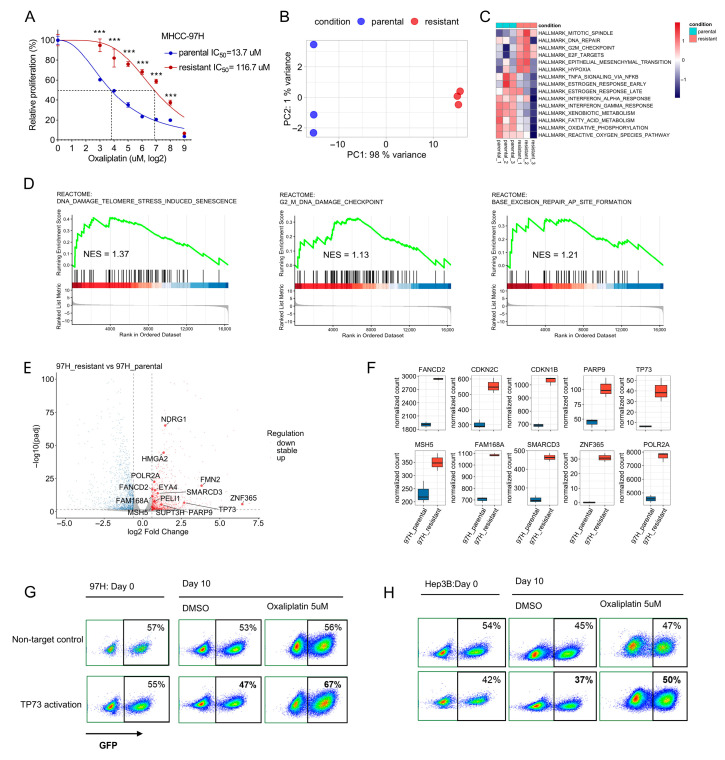
Establishment and transcriptomic profiling of an oxaliplatin-resistant MHCC-97H subline. (**A**) Dose–response curves and IC50 determination for parental and oxaliplatin-resistant MHCC-97H cells. Cells were treated with increasing concentrations of oxaliplatin for 72 h, and cell viability was measured using the CCK-8 assay. IC50 values were determined by nonlinear regression in GraphPad Prism v8.4.3 and are reported as mean ± SD (*n* = 3). Statistical comparison of IC50 values was performed using an unpaired two-tailed Student’s *t*-test; *p* values are shown. Significance is indicated as: ***, *p* < 0.001. (**B**) Principal component analysis of RNA-seq data (three independent biological replicates per condition) showing clear separation of resistant and parental samples. (**C**) GSVA (gene set variation analysis) of hallmark pathways comparing resistant versus parental MHCC-97H cells. (**D**) GSEA (gene set enrichment analysis) confirming significant enrichment of DNA damage response and repair gene sets in the resistant MHCC-97H subline relative to parental cells. Representative enrichment plots are shown. (**E**) Volcano plot of differential gene expression (resistant versus parental), with selected upregulated DNA damage response, checkpoint and stress-response genes highlighted. (**F**) Boxplots of DESeq2-normalized counts for selected differentially expressed DDR and cell-cycle genes across the three biological replicates per condition. (**G**,**H**) Representative flow cytometry dot plots for (**G**) MHCC-97H and (**H**) Hep3B from the competitive co-culture assay. GFP+ cells expressing either a non-targeting control (NT) or *TP73* CRISPRa constructs were mixed with GFP– wild-type cells, treated with oxaliplatin or vehicle, and analyzed by flow cytometry.

We generated an oxaliplatin-resistant derivative of the MHCC-97H hepatocellular carcinoma line by continuous exposure to low, sublethal concentrations of oxaliplatin. To quantify the degree of oxaliplatin resistance, dose–response curves were generated for the parental and oxaliplatin-resistant MHCC-97H lines following 72 h treatment. The resistant subline exhibited an IC50 of 116.7 μM (mean ± SD, *n* = 3) versus 13.7 μM for the parental line (mean ± SD, *n* = 3), corresponding to a resistance index of 8.5-fold (*p* < 0.001) (Figure 5A). Attempts to generate a resistant Hep3B subline using the same protocol were unsuccessful.

To define transcriptional programs associated with acquired resistance, we performed RNA-seq on parental and resistant MHCC-97H cells (three independent biological replicates per condition). Principal component analysis demonstrated clear separation of resistant and parental samples, consistent with a robust transcriptional reprogramming (Figure 5B). Pathway-level analysis by GSVA revealed upregulation in the resistant cells of hallmark programs including mitotic spindle, G2/M checkpoint, E2F targets and DNA repair, together with epithelial–mesenchymal transition; downregulated programs included interferon response and multiple metabolic gene sets (Figure 5C). Complementary GSEA confirmed enrichment of DNA damage response and repair gene sets in the resistant subline (Figure 5D).

At the gene level of DNA damage response and repair gene sets, the resistant MHCC-97H cells showed notable upregulation of genes, including the Fanconi anemia effector *FANCD2*, checkpoint/DDR modulators *PARP9* and *TP73*, and the recombination-associated gene *MSH5*. Concurrent increases in cell-cycle/transcriptional regulators (*CDKN2C*, *CDKN1B*, *POLR2A*, *HMGA2*) and stress-response genes such as *NDRG1* suggest coordinated remodeling of checkpoint control, transcriptional programs and stress responses in the resistant state (Figure 5E,F).

To test whether DDR-associated transcriptional changes observed in the oxaliplatin-resistant MHCC-97H subline are functionally relevant, we activated endogenous *TP73* using a CRISPRa approach in parental MHCC-97H and Hep3B cells and evaluated competitive fitness under oxaliplatin pressure. GFP+ cells expressing *TP73*-activating guides were mixed with GFP– wild-type cells (or with GFP+ cells expressing a non-targeting control), and cultures were treated with oxaliplatin. Following drug exposure, flow cytometry revealed a reproducible enrichment of GFP+ *TP73*-activated cells relative to the non-targeting control (Figure 5G,H). These data indicate that upregulation of *TP73* is sufficient to promote oxaliplatin resistance, supporting the functional relevance of transcriptomic DDR changes identified in the resistant subline.

### 3.6. Consistency Analysis Identifies DNA Damage Response Hits and Their Upregulation in HCC Tissues

**Figure 6:**
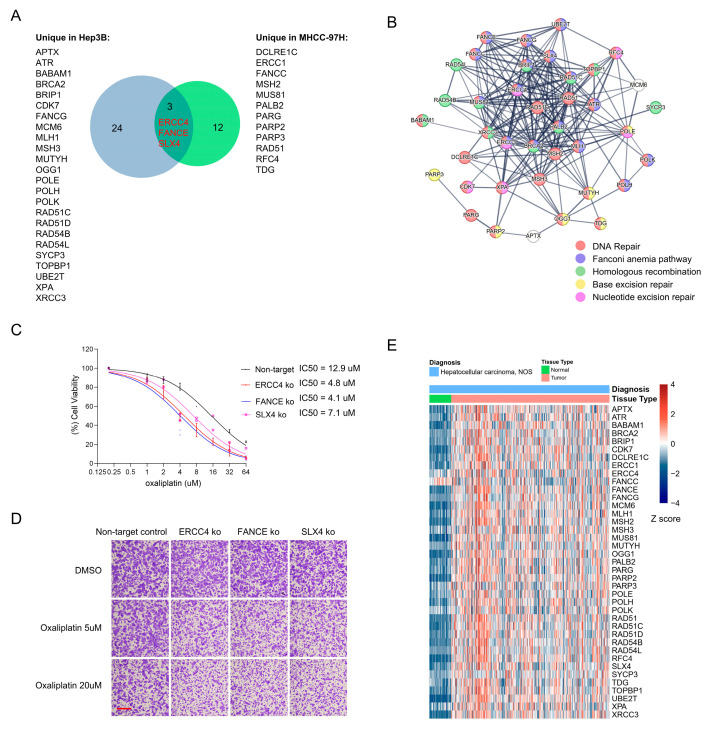
DNA damage response hits upregulated in HCC tissues. (**A**) Venn diagram summarizing overlap and cell line-specific DDR hits from the Hep3B and MHCC-97H CRISPR screens. Three genes (*ERCC4*, *FANCE*, *SLX4*) were common to both screens; 24 genes were Hep3B-specific and 12 were MHCC-97H-specific. Gene-level hits were defined as genes with ≥2 independent sgRNAs depleted in oxaliplatin-treated versus DMSO samples (log2 fold-change < −0.5 and *p*-value < 0.05). (**B**) STRING protein–protein interaction network and functional enrichment analysis performed on the union of Hep3B and MHCC-97H oxaliplatin sensitizers identified in (**A**). The network highlights convergence on DNA-repair processes, including Fanconi anemia, homologous recombination, base excision repair and nucleotide excision repair. Genes belonging to the indicated functional terms are color-coded. (**C**) Representative dose–response viability curves for MHCC-97H non-target control cells, knockouts of *ERCC4*, *FANCE* or *SLX4* following oxaliplatin treatment. Curves are representative of *n* = 3 independent biological experiments; IC50 values were calculated from these curves and are reported. (**D**) Representative crystal violet-stained plates showing survival of non-target control cells and gene-knockout MHCC-97H cells after oxaliplatin treatment (scale bar = 100 μm). (**E**) Heatmap of gene expression (log2[TPM + 1]) from TCGA-LIHC comparing tumor versus normal liver samples.

To assess the clinical relevance of DNA damage response hits from our CRISPR screens, we performed an integrative analysis of hits identified in Hep3B and MHCC-97H. A Venn analysis revealed three DDR genes that were consistently selected in both cell lines (*ERCC4*, *FANCE* and *SLX4*; Figure 6A, also identified in Figure 4F). Each cell line also yielded a set of unique DNA-repair candidates (24 Hep3B-specific and 12 for MHCC-97H -specific genes; Figure 6A). Despite these differences in composition, functional enrichment analysis using STRING showed that the two gene sets converge on related biological processes, including general DNA repair, the Fanconi anemia pathway, homologous recombination, base excision repair and nucleotide excision repair (Figure 6B).

To validate the three genes conserved across both screens, we generated CRISPR-mediated knockouts of *ERCC4*, *FANCE* and *SLX4* in the MHCC-97H HCC cell line and measured oxaliplatin sensitivity using dose–response viability assays. All three knockouts showed increased sensitivity to oxaliplatin, with significantly reduced IC50 values compared with control cells (Figure 6C). Consistent with the viability data, crystal violet staining of treated cultures revealed decreased survival of *ERCC4*-, *FANCE*- and *SLX4*-deficient cells relative to controls following oxaliplatin exposure (Figure 6D).

We next queried TCGA-LIHC expression data to determine whether these screen-identified genes are dysregulated in primary HCC. In cohort-level comparisons, a substantial proportion of both shared and cell line-specific DDR genes were upregulated in tumor samples relative to normal liver (Figure 6E). Analysis of 55 paired tumor-adjacent normal samples confirmed that most of these genes display higher expression in matched tumor tissues (Supplementary Figure S2). Together, these results indicate that multiple DNA-repair genes identified in vitro are also upregulated in clinical HCC, supporting their potential relevance as candidate biomarkers or therapeutic targets.

### 3.7. High Expression of DDR Genes Predicts Poorer Survival in Hepatocellular Carcinoma

**Figure 7:**
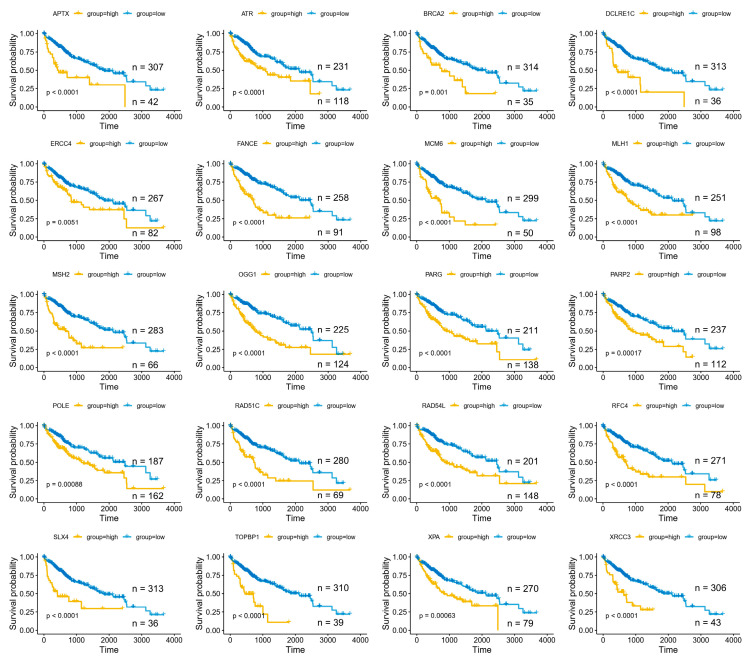
High expression of screen-identified DDR genes predicts poorer overall survival in HCC. Representative Kaplan–Meier overall-survival curves from the TCGA-LIHC cohort for selected DNA damage-response (DDR) genes identified in the CRISPR screens. For each gene, patients were stratified into high- and low-expression groups using an optimal cutpoint selected to minimize the log-rank *p*-value (best *p*-value) within the cohort; log-rank *p*-values are indicated on the individual plots.

We next evaluated whether the screen-identified DDR genes are associated with survival. Using an optimal-*p*-value cutpoint approach, analysis of the TCGA-LIHC cohort showed that elevated expression of most of these genes (37 of 39) were associated with significantly reduced overall survival. Notable examples include Fanconi anemia/ICL repair components (*FANCE*, *SLX4*, *ERCC4*/*XPF*), homologous recombination factors (*BRCA2*, *RAD51C*, *RAD54L*, XRCC3), base excision repair enzymes (*OGG1*, *APTX*), nucleotide excision repair components (*XPA*, *ERCC4*), mismatch repair genes (*MSH2*, *MLH1*), and replication/replication-stress regulators (*ATR*, *TOPBP1*, *MCM6*, *POLE*, *RFC4*) (Figure 7).

To avoid cutpoint selection bias, we analyzed the cohort using a predefined median expression split for each gene. With the median split, 23 of 39 genes (~59%) remained significantly associated with poorer overall survival (Supplementary Figure S3). We then performed Cox regression analyses to evaluate independence from available clinical covariates. In univariate Cox models, 31 genes were associated with prognosis. In multivariate Cox models that included age, sex, and tumor stage, gene *RAD54B* remained an independent predictor of overall survival (see Supplementary Figure S4).

To explore whether DDR expression correlates with clinical response to oxaliplatin-based HAIC, we analyzed bulk RNA-seq data from biopsy specimens collected from 36 HCC patients prior to initiation of HAIC. We compared expression of the 39 DDR genes identified from the CRISPR screen between patients who achieved an objective response (OR) and those who did not (non-OR). Several DDR genes were significantly upregulated in the non-OR group (Supplementary Figure S5, the original data can be found in Supplementary Files S1 and File S2), including *ATR*, *BRCA2*, *CDK7*, *MUS81*, *MUTYH*, *PARG*, *POLH*, *POLK* and *XPA*. These results are consistent with the hypothesis that elevated DDR gene expression may be linked to reduced sensitivity to HAIC, although they remain correlative.

## 4. Discussion

Effective systemic therapies for advanced HCC are limited. Oxaliplatin-based regimens, such as FOLFOX, have demonstrated clinical activity and improved survival in HCC, underscoring that oxaliplatin can be therapeutically meaningful in HCC [4,7]. However, intrinsic and acquired resistance to platinum agents limit their clinical benefit [14,17,24]. To explore strategies for improving oxaliplatin efficacy in HCC, we performed unbiased, genome-scale negative-selection CRISPR-Cas9 screens in two genetically distinct HCC cell lines under oxaliplatin exposure. Integrative analysis across screens identified DNA damage response (DDR) pathways—most notably, interstrand cross link (ICL) repair, nucleotide excision repair (NER) and the Fanconi anemia (FA) pathway—as conserved determinants of sensitivity to oxaliplatin.

DDR pathways are known to modulate platinum sensitivity in numerous tumor types; however, their role in HCC is less well characterized [17,25,26,27,28]. For example, elevated ERCC1 mRNA levels in ovarian cancer correlate with resistance to platinum-based chemotherapy [29,30], while homologous recombination-deficient breast and ovarian tumours display pronounced sensitivity to platinum compounds [31,32,33]. Conserved testicular germ cell tumors are uniquely susceptible to platinum agents in part due to impaired NER and robust apoptotic responses [34,35,36]. Our findings are therefore consistent with these broader paradigms and reinforce the conceptual rationale for targeting DDR to sensitize tumors to platinum compounds. Importantly, in our study, the translesion synthesis polymerase *POLH* and the NER factor *XPA* were validated to increased sensitivity to oxaliplatin at low concentrations (effective at both ~1 μM and 0.5 μM), suggesting that DDR perturbation might potentiate platinum cytotoxicity in a dose-sparing manner and thereby reduce treatment-related toxicity.

Transcriptomic profiling of an oxaliplatin-resistant MHCC-97H subline is consistent with a multifactorial resistance phenotype in which increased repair capacity, altered checkpoint control and broader transcriptional rewiring enable survival under chronic platinum stress [20,37,38,39,40]. The complexity of this phenotype underscores that, beyond DDR components, additional pathways may contribute to resistance and should be considered when designing combination therapies.

Oxaliplatin generates bulky platinum–DNA adducts and interstrand crosslinks that are tolerated or repaired in part by translesion synthesis (TLS) polymerases and the Fanconi anemia pathway. Consequently, pharmacologic suppression of these pathways provides a strong mechanistic rationale to potentiate oxaliplatin cytotoxicity and overcome lesion bypass. Several preclinical agents that target these nodes are already available and could be repurposed for combination strategies: (i) inhibitors of the REV1–REV7 interaction (e.g., the small molecule JH-RE-06) disrupt TLS scaffolding and have been reported to sensitize tumor cells to platinum agents in vitro; (ii) modulators of the FA pathway, such as the USP1 deubiquitinase inhibitor ML323, perturb FANCD2/FANCI regulation and increase sensitivity to interstrand-crosslinking chemotherapies; (iii) inhibitors of alternative polymerases and backup repair pathways, notably emerging POLθ inhibitors that preferentially target tumors with homologous-recombination defects, are attractive for combination approaches; and (iv) although selective POLH (Pol η) inhibitors are less clinically advanced, inhibition of TLS polymerases more broadly is a strategy to reduce lesion bypass.

More generally, several classes of DDR inhibitors, such as PARP, ATR/ATM, CHK1/CHK2 and WEE1 inhibitors, among others, offer rational combination partners for platinum agents [41,42,43,44]. PARP inhibitors are already approved or in advanced clinical development for tumors characterized by homologous recombination deficiency, including subsets of ovarian, breast, pancreatic and prostate cancers. However, BRCA-driven or broad homologous recombination deficiency phenotypes appear uncommon in HCC, and single-agent DDR inhibition has thus far shown limited benefit in unselected HCC. Against this backdrop, targeting the specific DDR dependencies uncovered (for example, NER, FA/ICL repair and TLS) in combination with oxaliplatin may be a more promising approach than monotherapy with current DDR inhibitors. Notably, TLS has recently emerged as a druggable tolerance pathway, and small molecules that disrupt TLS have been reported to sensitize cells to cisplatin and other platinum agents, providing an additional strategy to potentiate DNA-damaging therapies [45].

We acknowledge several limitations. First, our conclusions are principally derived from in vitro experiments and lack in vivo validation; consequently, their relevance to tumor growth and systemic treatment responses remains uncertain. Second, although the genome-scale screen and follow-up assays identified DDR genes that modulate oxaliplatin sensitivity, the downstream consequences of DDR gene ablation and the mechanisms by which sensitivity might be enhanced remain undefined. Third, comparisons between the generated resistant and wild-type MHCC-97H cell lines are correlative and descriptive, and individual findings were not systematically validated; confirmation in isogenic and patient-derived models is therefore required. Finally, our clinical associations are based on retrospective datasets and are thus susceptible to selection bias and confounding; prospective validation in independent cohorts is needed. Future work will prioritize in vivo validation, mechanistic rescue and pathway analyses, and evaluation in organoid/PDX models and prospective clinical cohorts. In particular, prospective functional validation using patient-derived organoids or ex vivo drug-sensitivity assays should be prioritized to determine whether DDR expression levels predict oxaliplatin resistance and to establish causality.

## 5. Conclusions

Our data suggests that conserved DDR components, notably NER and Fanconi anemia/ICL repair factors, modulate oxaliplatin response in HCC. These pathways represent candidate biomarkers and therapeutic targets whose inhibition may enhance oxaliplatin efficacy.

## Data Availability

The original data presented in the study are openly available in the NCBI Sequence Read Archive (SRA) at https://www.ncbi.nlm.nih.gov/sra/PRJNA1424547 (accessed on 27 February 2026; accession number PRJNA1424547). The HAIC RNA-seq data are available for download in the Supplementary Materials.

## Supplementary Materials

The following supporting information can be downloaded at: https://www.mdpi.com/article/10.3390/cancers18091360/s1, Figure S1: Oxaliplatin dose-response curves used to define CRISPR screen conditions. (A,B) Dose-response viability curves for (A) Hep3B and (B) MHCC-97H cells treated with increasing concentrations of oxaliplatin. Cell viability was measured after 48 h using the CCK-8 assay, normalized to vehicle control (0 μM), and plotted as percent viability. Nonlinear regression was used to fit dose-response curves. Data points represent mean ± SD from three independent experiments; Figure S2: Paired analysis of 55 tumors and adjacent normal sample pairs from TCGA-LIHC (log2[TPM + 1]); paired *t*-test results for individual genes are indicated in the panel. *p*-values are indicated in the figure; Figure S3: Kaplan–Meier analysis of DDR genes in the TCGA-LIHC cohort using a median expression split. Samples were dichotomized at each gene’s median expression into high (yellow) and low (blue) expression groups, and overall survival was compared by log-rank test (log-rank *p*-values are indicated on each plot). Across the 39 screen-identified DDR genes, 23 genes (~59%) were significantly associated with overall survival when grouped by median expression; The panels display the 12 genes with the strongest associations in median-split analyses. *p*-values are indicated in the figure; Figure S4: Forest plot of multivariate Cox models for genes identified as significant in univariate survival analysis (*p* < 0.05). The following genes were significant in univariate analysis: *RAD54B*, *FANCE*, *MCM6*, *MSH2*, *RAD54L*, *RAD51C*, *RFC4*, *UBE2T, DCLRE1C*, *RAD51*, *OGG1*, *TOPBP1*, *MUTYH*, *XRCC3*, *RAD51D*, *PARP2*, *ATR*, *PARG*, *CDK7*, *APTX*, *MUS81*, *TDG*, *BRCA2*, *PALB2*, *MLH1*, *ERCC1*, *SLX4*, *FANCG*, *POLE*, *BRIP1*, and *XPA*. The multivariate model displayed here includes these genes together with clinical covariates (age, sex, and AJCC pathological T stage). Points indicate hazard ratios (HR) and horizontal lines denote 95% confidence intervals (log scale); the red dashed vertical line marks HR = 1. The red star highlights RAD54B, which remained an independent predictor of overall survival in the multivariate analysis (*p* < 0.05); Figure S5: DDR gene expression of biopsy specimens collected from 36 HCC patients prior to initiation of HAIC. Boxplots show transcript abundance (TPM) for the 39 DDR genes identified in the CRiSPR screen; each point represents an individual biopsy (*n* = 36). Samples from non-responders (non_OR) are shown in green and responders (OR) in orange. *p*-values for the OR versus non-OR comparison are indicated above each gene; genes with *p* < 0.05 are highlighted with red boxes. The red-boxed genes significantly downregulated in the OR group are: *ATR*, *BRCA2*, *CDK7*, *MUS81*, *MUTYH*, *PARG*, *POLH*, *POLK* and *XPA*. *p*-values are indicated in the figure; Figure S6: Quantification by densitometry of Cas9 levels detected by western blotting as shown in Figure 1A (left) and Figure 4A (right). Data are presented as mean ± SD (*n* = 3); Figure S7: Original (uncropped) western blot images for β-actin in 97H and Hep3B cells. Lanes (left to right): 97H, 97H + Cas9, Hep3B, Hep3B + Cas9. (A) Colorimetric images. (B) Chemiluminescent images. (C) Merged images of (A,B), shown to allow simultaneous visualization of bands and molecular-weight markers; Figure S8: Original uncropped western blot images for Cas9 in 97H and Hep3B cells. Lanes (left to right): 97H, 97H + Cas9, Hep3B, Hep3B + Cas9 cells. (A) Colorimetric images. (B) Short-exposure chemiluminescent images. (C) Long-exposure chemiluminescent images. (D) Merged images of (A,C) to allow simultaneous visualization of bands and molecular-weight markers; Figure S9: Original uncropped Western blot images for Figure 3. Left: original chemiluminescent images; middle: colorimetric images; right: merged images (chemiluminescent overlaid on colorimetric images) to allow simultaneous visualization of bands and molecular-weight markers; File S1: metaHAIC; File S2: TPM-HAIC.

## Abbreviations

The following abbreviations are used in this manuscript:

BER	Base excision repair
EMT	Epithelial–mesenchymal transition
FA	Fanconi anemia
FOLFOX	Folinic acid (leucovorin), fluorouracil and oxaliplatin regimen
GeCKO	Genome-scale CRISPR Knock-Out library (GeCKO)
GSEA	Gene set enrichment analysis
GSVA	Gene set variation analysis
HCC	Hepatocellular carcinoma
HAIC	Hepatic artery infusion chemotherapy
HR	Homologous recombination
IC50	Half-maximal inhibitory concentration
ICL	Interstrand cross-link
MOI	Multiplicity of infection
NER	Nucleotide excision repair
PARP	Poly(ADP-ribose) polymerase
TLS	Translesion synthesis
