# Supplementary figures and images for: Genome-Scale CRISPR Screens Reveal DNA Repair Dependencies That Sensitize Hepatocellular Carcinoma to Oxaliplatin
Source: Cancers (Basel). 2026 Apr 24;18(9):1360. doi: 10.3390/cancers18091360 (PMC13162874; doi:10.3390/cancers18091360)

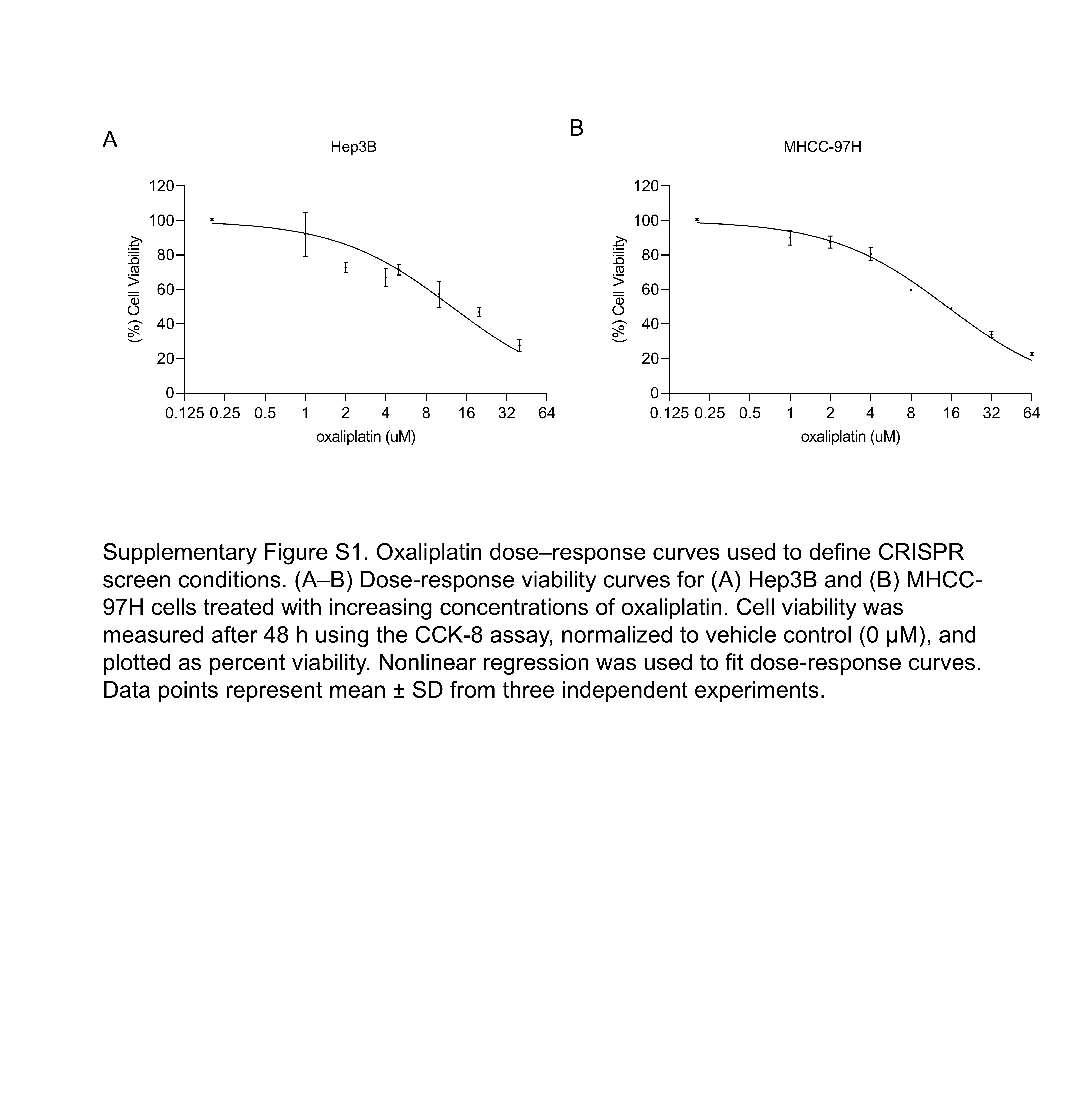

Supplement: Supplementary file 1 [file cancers-18-01360-s001.zip › Supplementary Figure S1.TIF]

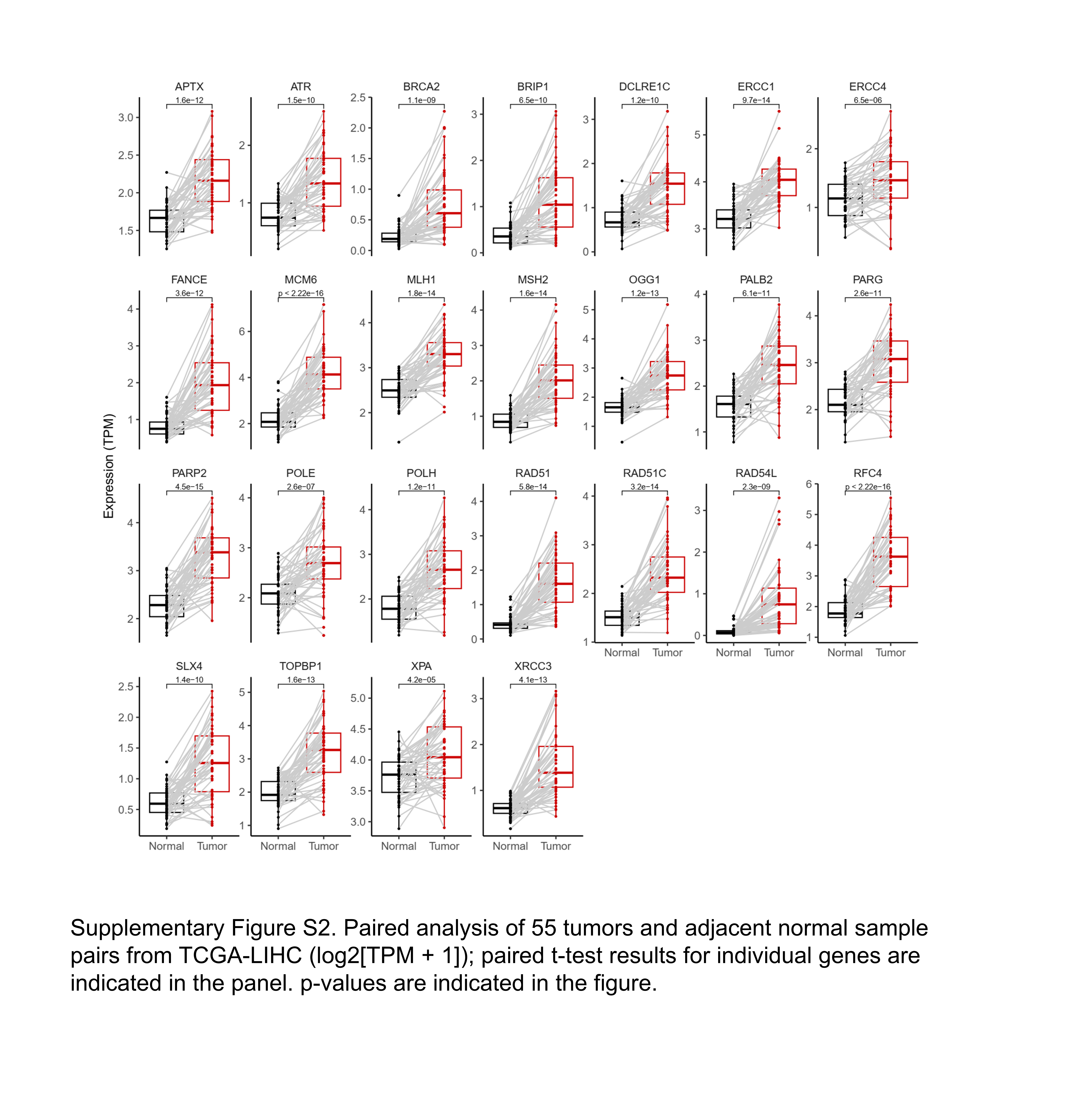

Supplement: Supplementary file 1 [file cancers-18-01360-s001.zip › Supplementary Figure S2.TIF]

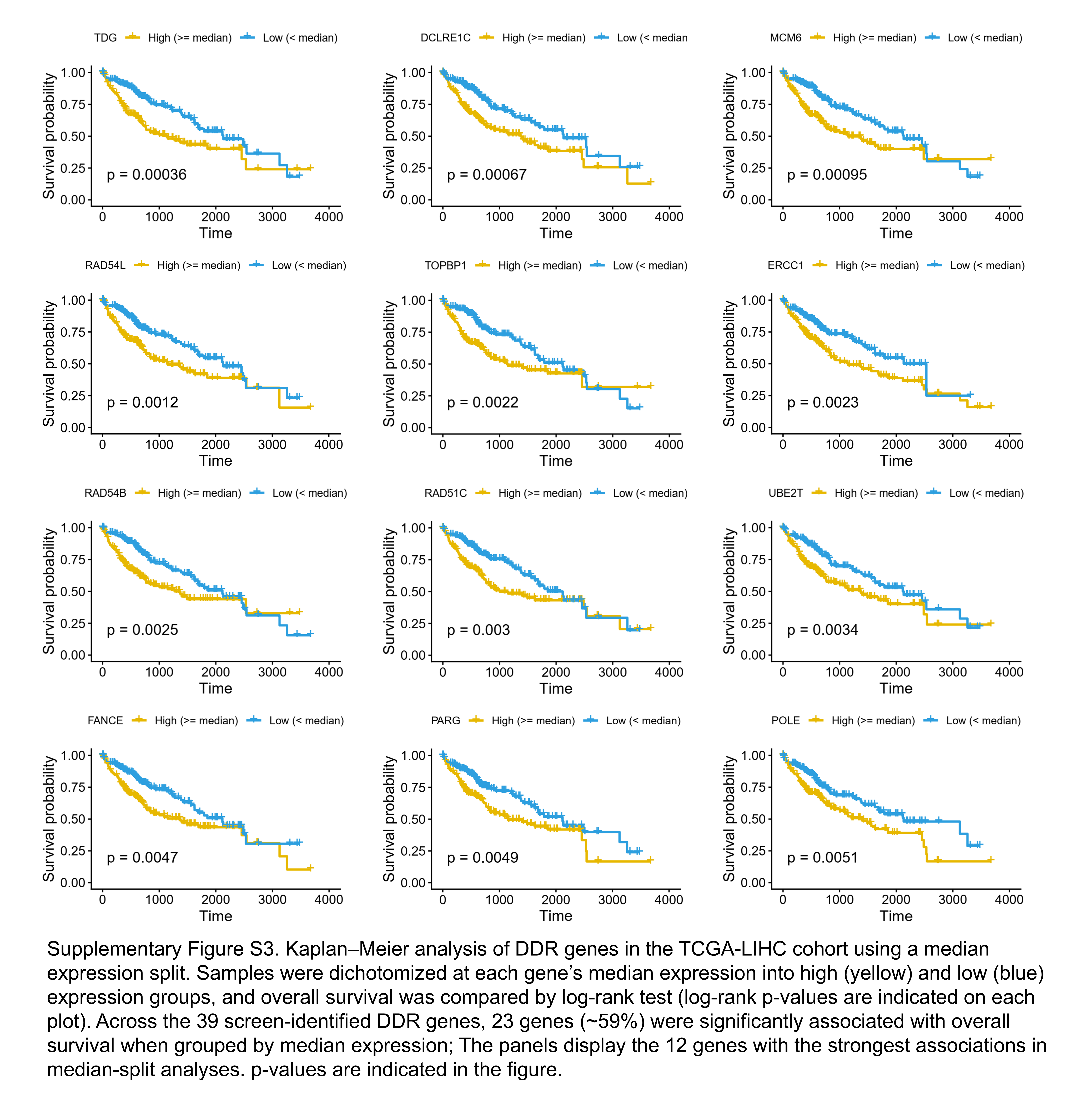

Supplement: Supplementary file 1 [file cancers-18-01360-s001.zip › Supplementary Figure S3.TIF]

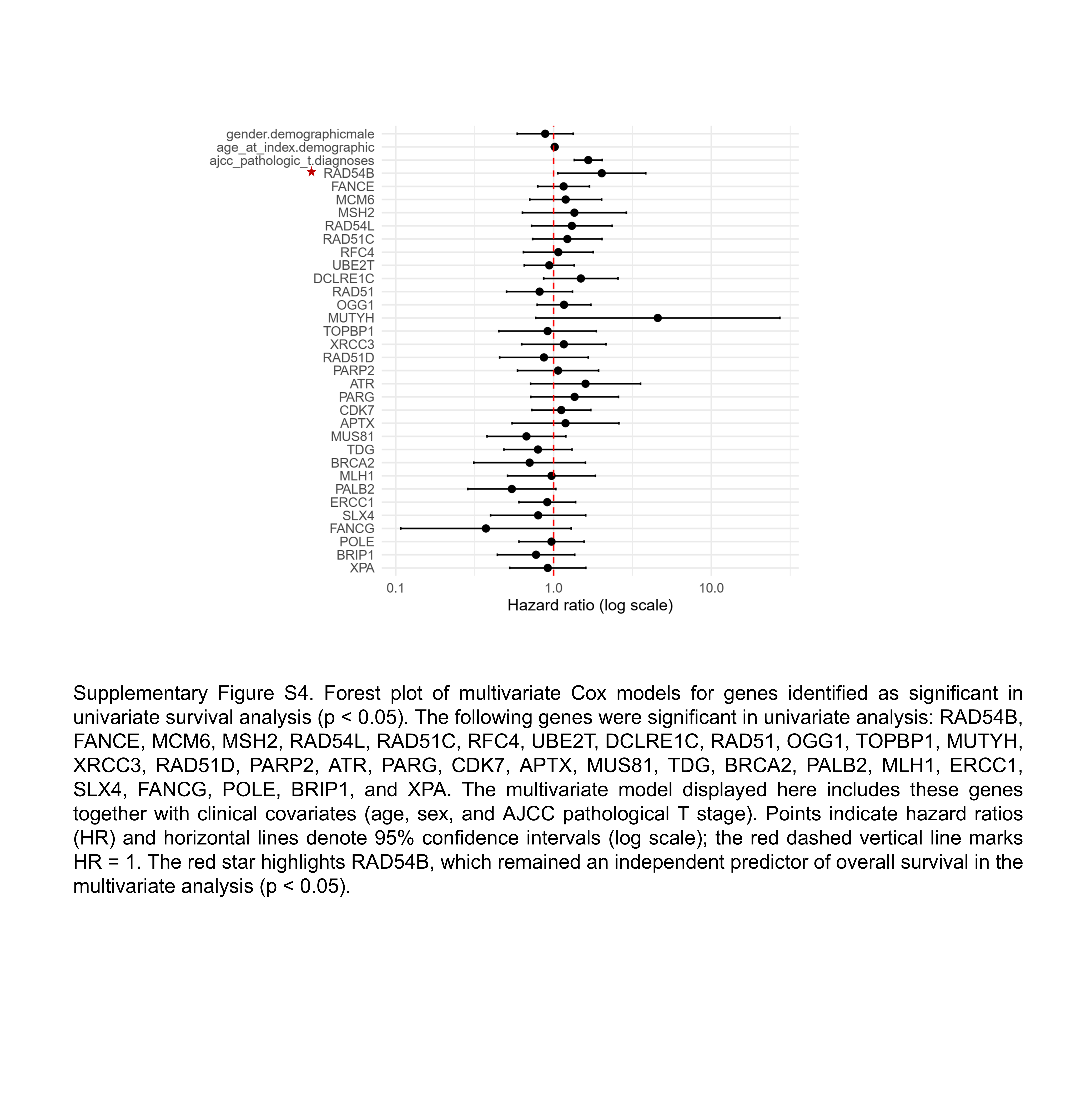

Supplement: Supplementary file 1 [file cancers-18-01360-s001.zip › Supplementary Figure S4.TIF]

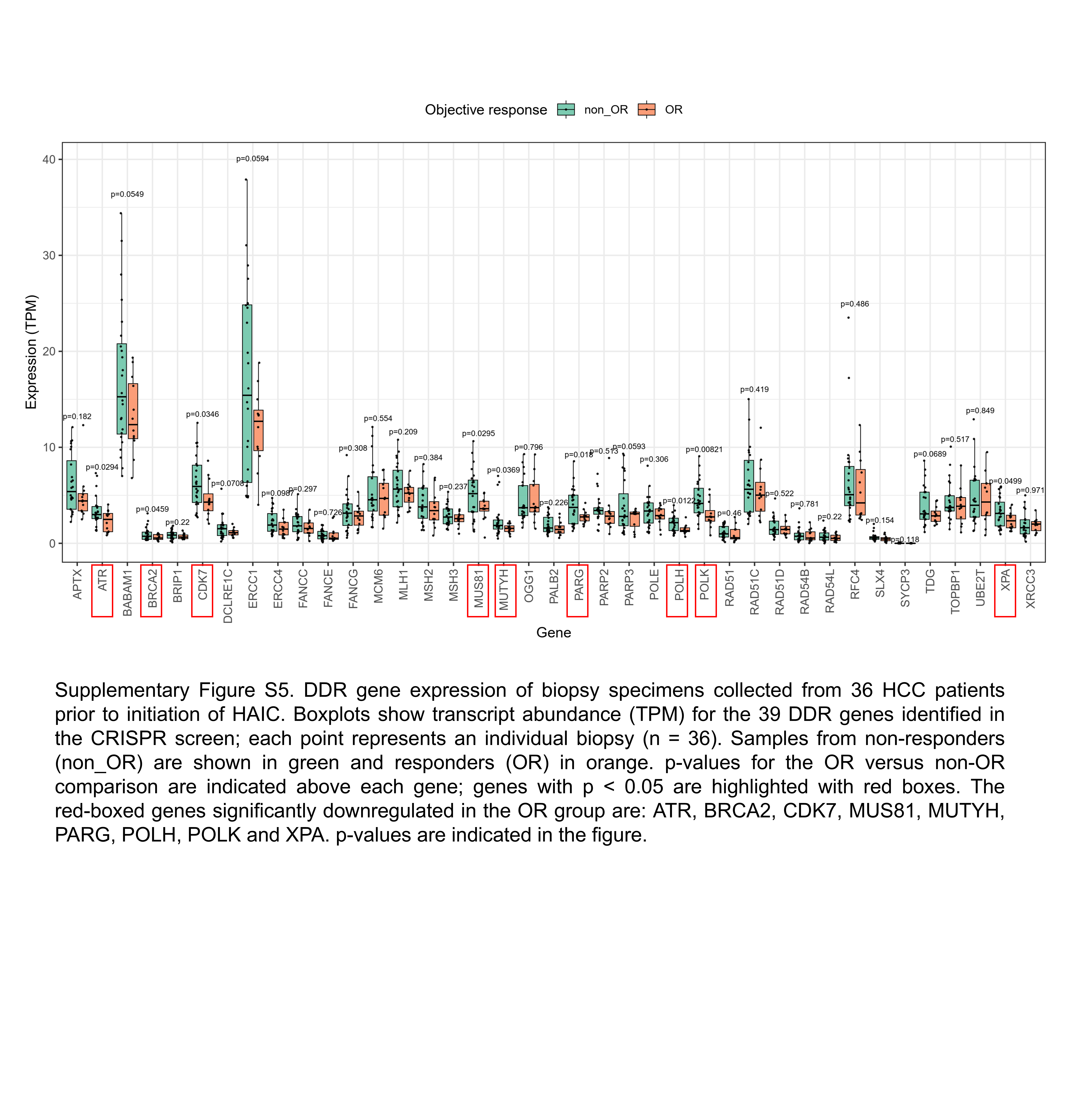

Supplement: Supplementary file 1 [file cancers-18-01360-s001.zip › Supplementary Figure S5.TIF]
